# The Death to Onchocerciasis and Lymphatic Filariasis (DOLF) project: Accomplishments and ongoing research

**DOI:** 10.1371/journal.pntd.0013953

**Published:** 2026-02-03

**Authors:** Peter U. Fischer, Christopher L. King, Philip J. Budge, Gary J. Weil, on behalf of the DOLF Consortium

**Affiliations:** 1 Division of Infectious Diseases, Department of Medicine, Washington University School of Medicine, St. Louis, Missouri, United States of America; 2 Center for Global Health and Diseases, Department of Pathology, Case Western Reserve University School of Medicine, Cleveland, Ohio, United States of America; Institute of Continuing Medical Education of Ioannina, GREECE

## Abstract

The Death to Onchocerciasis and Lymphatic Filariasis (DOLF) project conducts clinical and translational research to accelerate the global elimination of lymphatic filariasis (LF) and onchocerciasis (river blindness). Research needs were identified during several meetings of experts and helped study design. Over the last 15 years the project has conducted mass drug administration (MDA) studies and clinical trials with outstanding research teams in 11 countries that have led to many important discoveries. For example, DOLF studies showed that annual MDA was as effective as semiannual MDA for LF elimination and that semiannual MDA with albendazole alone can eliminate LF in areas where ivermectin cannot be safely used. Multiple studies showed that a single dose of the triple-drug combination ivermectin plus diethylcarbamazine and albendazole (IDA) was well tolerated and more effective than previously recommended treatments for LF. Results of these studies led the World Health Organization (WHO) to endorse IDA for LF elimination programs in countries without coendemic onchocerciasis or loiasis. More recently, we have shown that single-dose treatment with moxidectin plus albendazole is superior to the ivermectin plus albendazole regimen currently recommended for LF elimination in Africa. Safety and efficacy studies of combination treatments for onchocerciasis have shown that IDA is more effective against adult *Onchocerca volvulus* worms than ivermectin plus albendazole, although the effect was modest. We are currently testing the macrofilaricidal efficacy of other drug combinations and treatment schedules. DOLF researchers have also conducted important studies that are not clinical trials. For example, we discovered that some people with high *Loa loa* microfilaria (Mf, first-stage larvae) densities have cross-reactive filarial antigen tests. DOLF has also addressed the issue of MDA compliance, because improved treatments cannot be effective if people do not swallow the tablets. We highlight here some of DOLF’s important discoveries that support the control and elimination of neglected tropical diseases caused by helminths.

## Introduction

The Death to Onchocerciasis and Lymphatic Filariasis (DOLF) project is a large research program that conducts clinical and translational research to test new treatments to support efforts to eliminate two major neglected tropical diseases, namely lymphatic filariasis (LF) and onchocerciasis. The project arose following several meetings of experts convened by the Bill & Melinda Gates Foundation (now Gates Foundation) in 2009 that identified and prioritized key technical challenges faced by the Global Programme to Eliminate Lymphatic Filariasis (GPELF). The meetings also considered similar issues regarding onchocerciasis, because intervention was shifting its goal from control to elimination at that time. Our team was asked to develop and implement a research program based on results of the scoping exercise meetings. We focused initially on key questions that might be answered by field research over five years. Some 10 years after its initiation, GPELF had already made good progress. However, problems had been identified that might be solved by intensified intervention research [1,2]. Furthermore, the progress of the onchocerciassis elimination program of the Americas and results from some onchocerciasis foci in Africa fueled the discussion about elimination of onchocerciasis in Africa [3–6]. The studies proposed by DOLF aimed to increase the chances of onchocerciasis elimination in Africa.

The DOLF project was funded in November 2009. DOLF’s lead scientific and administrative core at Washington University in St. Louis was asked to coordinate (and sometimes conduct) North-South and South-South research collaborations to replace hunch and conjecture with reliable data. At that time, the main tool employed by elimination programs for LF and onchocerciasis in disease-endemic areas was mass drug administration (MDA) of donated antifilarial medications without screening individuals for infection [7]. The purpose of the DOLF project was to conduct field research to test novel treatments and treatment strategies that might accelerate the elimination of these major neglected tropical diseases. By design, our studies focused on novel therapies with existing, registered drugs, because other research groups were working to develop and test novel compounds.

At the beginning of the elimination programs, annual MDA with ivermectin was recommended for onchocerciasis and two-drug regimens (ivermectin plus albendazole where onchocerciasis is co-endemic or diethylcarbamazine plus albendazole in other areas) were recommended for LF [7,8]. When DOLF started in 2010, 18 of the 81 countries that were endemic for LF in the year 2000 had not yet started MDA and full geographical coverage had not been achieved in the five countries with the highest LF burden (Bangladesh, the Democratic Republic of the Congo, India, Indonesia and Nigeria) [9,10]. Intervention for onchocerciasis in Africa was still focused on morbidity control in 2010, targeted mostly hyper- and mesoendemic areas, and elimination was only recommended for selected areas where it was considered to be feasible [11].

In this review, we have summarized the general DOLF approach and described major research outcomes along with their policy implications for disease elimination programs. While our studies have identified and validated important new treatment regimens, DOLF studies have also contributed important outcomes in the areas of social science. That is because better treatment regimens do not work if people do not swallow the medications! Therefore, from the beginning, social science research was included to help strengthen compliance with mass drug administration [12]. This review also describes unexpected or off-target advances that have come out of our field studies. These surprises are examples of the exciting dividends that occur when people with open eyes and minds are actively engaged with people impacted by Neglected Tropical Diseases (NTDs). Recognizing that more work is needed, we have also outlined ongoing projects and planned next steps for DOLF.

**General approach and initial questions addressed:** The scoping exercises generated a long list of potential projects. We worked with the Gates Foundation and external experts to prioritize topics and studies based on potential impact for disease elimination programs, lack of duplication by other research groups, and the likelihood of obtaining useful results within the 5-year funding period of the grant. The initial projects are listed in Box 1.

**Box 1. Initial focus areas for DOLF in 2009**

1. Annual versus semiannual MDA for LF and onchocerciasis
2. MDA for elimination of LF in areas with coendemic loaisis
3. Effects of community MDA for LF and onchocerciasis on soil-transmitted helminth (STH) infection prevalence and intensity
4. Clinical development of improved treatments for LF
5. Clinical development of macrofilaricidal treatments for onchocerciasis

Many countries had initiated MDA for LF and onchocerciasis before 2010, and infection parameters in endemic areas at that time were often unknown. Our own pilot surveys found that infection prevalence data in national or international registries were often inaccurate. Our initial steps for these projects included identification of potential study sites with sufficient disease endemicity, reasonable political stability, and reliable endemic-country partners with the capacity to conduct the studies. In many cases, pilot surveys were conducted to identify suitable study sites. Results from some but not all of these pilot surveys were published. For example, in Gabon, 856 people from 12 villages with reported circulating filarial antigen prevalences >20% were screened for *W. bancrofti* microfilariae (Mf) in night blood, but no Mf-positive individuals were detected [Boussinesq and colleagues 2011, personal communication]. In Nigeria, a convenience sample of 643 people from 2 districts were screened for *O. volvulus* nodules, but microfiladermia prevalences and densities were too low for clinical trials [13]. In some cases, potential country collaborators had to perform extensive data reviews before study sites were selected [14,15]. After identifying a potential study site, preliminary site visits were performed to assess the existing facilities, personnel, and possible requirements for training, research infrastructure improvements, and major equipment (vehicles). Duplicate study sites were sometimes included to make the study outcomes more robust and because some studies were at risk for political unrest or war. Preliminary visits and pilot surveys helped to establish good working relationships between DOLF and endemic-country scientists and institutions. Partner institutions included ministries of health (MOH), universities, and/or research institutes that had good working relationships with their respective MOH and regulatory authorities (ethical review committees and drug regulatory authorities).

After identifying study sites and partners, the next steps included writing or modifying protocols according to local conditions, development of budgets and contracts, ethical approval, clinical trial registration, data management plans, and publication agreements. Internet connectivity was required for all studies to facilitate communication and data transfer. Some study sites required significant upgrades to existing communication capabilities, while others required more significant investments such as installation of cellular towers or satellite internet. We also hired certified site monitors, purchased clinical trial insurance and established data safety and monitoring boards for all randomized clinical trials. When infrastructure improvements were in place, we held project initiation meetings to train personnel on protocols, good clinical practice, and standard operating procedures for the study.

DOLF closely monitored the progress of field projects and made interim adjustments as needed. Data managers in St. Louis worked with counterparts in country to organize and clean study data. Manuscript preparation was a joint effort with endemic-country scientists listed as first and/or last authors for most papers.

DOLF periodically convened meetings of advisors (a “technical advisory team” or TAT) to review early results and propose next steps. The TAT included experts from academia, industry, national NTD programs, and international organizations. Interim results sometimes led to our doubling down on some projects and dropping others based on recommendations from the TAT and Gates Foundation program officers. The Gates Foundation practiced “adaptive grant management” for this research program and provided additional funding for follow-up studies based on exciting results obtained during the first years of DOLF. Supplemental funding was sometimes necessary to support progression of an initial promising discovery through confirmatory research followed by safety and acceptability studies required for policy change.

## Results

DOLF research partner institutions and major projects are listed in Box 2.

**Box 2. Major DOLF resevarch partners, key contact person, and consortium members^1^.**

**Table:** 

Institutions (representative)	Country	Main Project(s) (Objective identifiers)
Washington University School of Medicine, St. Louis, Missouri (Gary J. Weil, Peter U. Fischer, Philip J. Budge)	USA	Secretariat and DOLF headquarters (1-7)
Case Western Reserve University, Cleveland, Ohio(Christopher L. King, Catherine M. Bjerum)	USA	Clinical trials for LF and onchocerciasis in PNG, Côte d’Ivoire, and Ghana; Community MDA trials in PNG and Côte d’Ivoire (1-4)
Centers for Disease Control and Prevention, Atlanta, Georgia (Christine Dubray)	USA	Community trial of the safety and efficacy of IDA vs DA for LF in Haiti. (4.1)
Medicines Development for Global Health (MDGH) (Sally Kinrade)	Australia	Community trial to compare the tolerability of IA vs MoxA in an area endemic for onchocerciasis in Côte d’Ivoire (4.3,5.2)
Tropical Diseases Research Group, Murdoch Children’s Research Institute, Melbourne, Victoria (Andrew C. Steer)	Australia	Community trial of the safety and efficacy of IDA vs DA for LF in Fiji (4.1)
Bruyère Research Institute, Ottawa; School of Epidemiology and Public Health, University of Ottawa, Ottawa, (Alison Krentel)	Canada	Community studies of the acceptability of MDA in many countries (4.1,4.2)
Centre Suisse de Recherches Scientifiques (CSRS) en Côte d’Ivoire, Abidjan (Benjamin G. Koudou)	Côte d’Ivoire	Once vs twice yearly community trial of annual vs semiannual MDA for onchocerciasis and LF elimination; Clinical trials of IDA vs IA, semiannual albendazole monotherapy, and moxidectin combination therapies for LF; Community trial to compare the tolerability of IA vs MoxA in an area endemic for onchocerciasis. (1,3,4.3,6.2,6.3)
Programme National de Lutte contre les Maladies Tropicales Négligées à Chimiothérapie Préventive, Ministère de la Santé Publique, Kinshasa (Naomi Awaca-Uvon)	Democratic Republic of the Congo	Community trial of semiannual albendazole for LF and STH (2)
Fiji Ministry of Health and Medical Services, Suva (Josaia Samuela)	Fiji	Community trial of the safety and efficacy of IDA vs DA for LF (4.1)
Institut de Recherche pour le Développement, INSERM Unité, Université de Montpellier, Montpellier (Michel Boussinesq)	France	Community trials of semiannual albendazole for LF and STH Community trials in the Republic of Congo and in the Democratic Republic of the Congo (2,3)
Institute for Medical Microbiology, Immunology and Parasitology, University Hospital Bonn, Bonn (Achim Hoerauf)	Germany	Clinical trial of semiannual vs annual treatment with ivermectin or ivermectin plus albendazole for onchocerciasis in Ghana; Histological analysis of *Onchocerca* nodules from clinical trials (5)
Fred Newton Binka School of Public Health, University of Health and Allied Sciences, Ho (Nicholas Opoku)	Ghana	Clinical trial of semiannual vs annual treatment with ivermectin or ivermectin plus albendazole for onchocerciasis; Clinical trial of different combination treatments (IA, IDA, 3 doses of IDA) for onchocerciasis (5)
Kwame Nkrumah University of Science and Technology, Kumasi (Alex Debrah)	Ghana	Clinical trial of semiannual vs annual treatment with ivermectin or ivermectin plus albendazole for onchocerciasis (5)
Ministère de la Santé et de la Population, Port-au-Prince (Jean Frantz Lemoine)	Haiti	Community trial of the safety and efficacy of IDA vs DA for LF (4.1)
ICMR-Vector Control Research Centre, Puducherry (Purushothaman Jambulingam)	India	Community trial of the safety and efficacy of IDA vs DA for LF (4.1)
Department of Parasitology, Faculty of Medicine, Universitas Indonesia, Jakarta (Taniawati Supali)	Indonesia	Community trial of annual vs semiannual MDA with DEC plus albendazole for LF and STH; Clinical trials of IDA vs DA for LF; Community trial of the safety and efficacy of IDA vs DA for LF (1,3,4.1,4.2,6.2)
Liberian Institute for Biomedical Research/National Public Health Institute of Liberia, Charlesville/Monrovia (Fatorma Bolay†, Patrick Kpanyen)	Liberia	Community trial of annual vs semiannual MDA for onchocerciasis, LF and STH; Clinical trial of four different combination treatments (IA, IDA, MoxA, MoxDA for onchocerciasis (1,3,5.2,6.1,6.2,6.3)
Department of Public Health, Erasmus MC, University Medical Center Rotterdam, Rotterdam (Wilma Stolk)	Netherlands	Computer simulation modeling of the impact of annual vs semiannual MDA for LF elimination (1)
Department of Infection and Immunity, Papua New Guinea Institute of Medical Research, Goroka and Madang (Moses Laman)	Papua New Guinea	Community trial of annual vs semiannual MDA with DEC plus albendazole for and STH; Clinical trials of IDA vs DA for LF; Community trial of the safety and efficacy of IDA vs DA for LF (1,3,4.1,4.2)
Ministère de la Santé et de la Population, Brazzaville (Francois Missamou)	Republic of Congo	Community study of semiannual albendazole for LF elimination and STH (2,3)
Ministry of Health, Anti-Filariasis Campaign, Colombo (Sandhya D. Samarasekera); University of Ruhuna, Galle (Channa Yahathugoda)	Sri Lanka	Xenomonitoring and comprehensive assessment of LF elimination; Evaluation of filarial diagnostic tests (7)

^1^Pilot surveys in Gabon, Mozambique, Nigeria, and Tanzania did not identify suitable study sites.

### 1. Annual vs semiannual MDA for LF and onchocerciasis

One potential strategy to accelerate elimination of onchocerciasis and LF is more frequent delivery of MDA. Semiannual or quarterly MDA with ivermectin has been used in the Americas and in several isolated foci in Africa to eliminate onchocerciasis [16,17]. The potential added value of semiannual MDA for LF elimination was unknown in 2009. DOLF sponsored a simulation modeling and cost projection study that was based on data from India and West Africa. Briefly, that study suggested that more frequent MDA should accelerate LF elimination and reduce the overall cost of programs [18]. DOLF tested this hypothesis with field studies in Indonesia, Papua New Guinea, Côte d’Ivoire, and two sites in Liberia. These showed that annual MDA was as effective for reducing Mf and filarial antigen prevalence as semiannual (twice yearly) MDA [19–22].

**Significance:** These results from different settings and countries suggest that national LF elimination programs should focus on providing one high-quality round of MDA per year and not strain limited resources by trying to provide two rounds per year. These results were considered when WHO decided against recommending twice-yearly MDA for LF elimination in its 2017 guidelines [23]. For onchocerciasis, results from DOLF’s studies in West Africa were less clear because they were not designed to document subtle differences in impact on skin Mf. Twice-yearly MDA could be as effective or even more effective than annual MDA to reduce transmission [19,20]. However, high community uptake of MDA is crucial for both onchocerciasis and LF control and elimination. Semiannual MDA is unlikely to provide a significant advantage over annual MDA for eliminating onchocerciasis in settings with low compliance (percentage of targeted people who swallow tablets) and especially in areas with a high number of people who are systematically non-compliant (so-called “never treated” individuals).

### 2. MDA for elimination of LF in areas with coendemic loiasis

The strategy of using MDA with ivermectin to eliminate onchocerciasis or ivermectin plus albendazole to eliminate LF is problematic in areas that are co-endemic for loiasis, because ivermectin can cause serious adverse events in individuals with high *L. loa* Mf densities. The risk of severe or serious adverse events after ivermectin is higher in persons with high *L. loa* blood Mf counts [24]. A single dose treatment with albendazole has no direct microfilaricidal effect on *W. bancrofti* Mf, but it has embryostatic properties on adult worms [25]. Therefore, we designed a number of clinical and community trials of albendazole monotherapy for bancroftian filariasis. A clinical trial in Côte d’Ivoire compared the effect of 3 annual doses of ivermectin 200 µg plus 400 mg of albendazole with 6 semiannual doses of albendazole alone (either 400 or 800 mg) showed that all regimens cleared *W. bancrofti* microfilaremia and had a partial macrofilaricidal effect [26]. Large, long-term community trials in the Republic of Congo and the Democratic Republic of the Congo also showed that MDA with semiannual albendazole (400 mg) cleared *W. bancrofti* Mf in *L. loa* co-endemic countries [27–30]. Further analysis of data from these studies showed a strong correlation between individual compliance with MDA and treatment efficacy [31,32].

**Significance:** These studies in multiple countries provided strong evidence that semiannual albendazole is effective for treating bancroftian filariasis and that MDA with albendazole can be safely used to eliminate LF in countries with coendemic loiasis. These studies represent an example of DOLF research providing evidence for changes in policy to support disease elimination, and they contributed to WHO’s recommendations for alternative MDA approaches to eliminate LF in Africa [23].

### 3. Effects of community MDA for LF and onchocerciasis on STH prevalence and intensity

DOLF’s large community trials of the effects of MDA on LF have provided some of the most detailed information on the impact of MDA regimens used for LF elimination on soil-transmitted helminth infections (STH). Regimens studied included ivermectin plus albendazole, DEC plus albendazole, and albendazole alone. Results of these studies showed that the sustained reduction and potential clearance of STH depend on many factors, including STH species, the baseline prevalence, the drug(s) used for MDA, MDA frequency and compliance, and general water, sanitation, and hygiene (WASH) conditions. For example, in an area in the Republic of Congo with a baseline low prevalence hookworm, it was possible to locally eliminate the infection with twice-yearly albendazole [27,29,30]. A similar trend was observed in a parallel study in the Democratic Republic of Congo in an area with higher baseline hookworm prevalence where prevalence decreased from 50% to 20% after 6 semiannual rounds of albendazole. This intervention resulted in a marked reduction in the prevalence of heavy and moderate density STH infections [28]. A similar reduction in hookworm prevalence and egg counts was also observed in a study in Côte d’Ivoire after several rounds of MDA with ivermectin plus albendazole. However, annual and semiannual MDA were equally effective for treating hookworm in that study [33]. Similar results were obtained regarding hookworm and other STH in two large community MDA studies in different regions of Liberia (in Lofa and Harper counties) with equivalent reductions in STH prevalence and egg counts after three or five rounds of annual or semiannual MDA [19,20]. Because of the high prevalence of intestinal schistosomiasis in Lofa County, praziquantel was also provided as MDA, but the effect on *S. mansoni* prevalence and density of infection one year after the last round of MDA was negligible [20].

DOLF also supported a study comparing the impact of annual versus semiannual MDA with DEC plus albendazole on hookworm infections in highly endemic areas in Papua New Guinea. First results indicated that two rounds of MDA were required to reduce hookworm prevalence and intensity of infection. Thus, semi-annual MDA achieved this in one year, whereas it took two years to achieve a similar reduction with annual MDA. Notable was that only semiannual MDA increased hemoglobin levels in men. A separate DOLF MDA study in Papua New Guinea showed that a single dose of the triple drug combination IDA was more effective against hookworm and strongyloidiasis than DEC plus albendazole [34]. Both MDA therapies reduced hookworm transmission, which was more pronounced with IDA.

**Significance:** These studies in different settings and countries clearly show that MDA for LF and/or onchocerciasis reduces STH prevalence and intensities. However, the impact of MDA in the time frame of these studies varied somewhat by location and STH species, with greater effects against hookworm, variable effects against trichuriasis, and little lasting impact on ascariasis. Semiannual MDA was not superior to annual MDA in most of these studies, with a possible exception in areas with high hookworm burdens, such as PNG. Annual or semiannual MDA will not eliminate STH in most settings. The relatively short duration of our studies does not allow us to predict the duration of the effects of MDA for filarial infections on STH after MDA is stopped. However, we believe that MDA for filarial infections can serve as induction therapy for control of STH in communities by resetting prevalence and intensities of infections to levels that can be maintained by deworming programs that focus on school-aged and preschool children.

### 4. Clinical development of improved treatments for LF

#### 4.1. IDA triple drug treatment for LF.

DEC combined with albendazole is recommended to eliminate LF in countries that are not co-endemic for onchocerciasis, while ivermectin plus albendazole is recommended in countries with onchocerciasis [7]. Before the first DOLF studies, the safety and efficacy of co-administration of all three drugs had never been tested. The first DOLF pilot trial of IDA (ivermectin plus DEC plus albendazole) in Papua New Guinea (PNG) showed that all 12 individuals with *W. bancrofti* who were treated with single dose of the drug combination were Mf negative one year after treatment, whereas 11 of 12 individuals treated with DEC plus albendazole were Mf positive at that time [35]. Subsequent clinical trials in PNG and Côte d’Ivoire (with ivermectin plus albendazole as the comparator treatment) confirmed that IDA was well tolerated and superior to the two-drug regimens [36,37]. Although IDA was only partially macrofilaricidal, the clearance of Mf after IDA treatment lasted for at least 5 years in most people [38]. Pharmacokinetic studies of IDA showed no significant drug-to-drug interactions of concern [35,39]. IDA also effectively cleared Mf of *Brugia timori* and *B. malayi* [40,41].

The superior efficacy of IDA over the previously recommended two-drug regimens suggested that IDA should be considered for use in countries that had previously used DEC plus albendazole as their MDA regimen for LF elimination. IDA cannot be safely used as MDA in areas coendemic for LF and onchocerciasis (where ivermectin plus albendazole is currently recommended), because DEC can cause serious ocular adverse events in individuals with onchocerciasis [42].

Computer modelling studies predicted that substitution of IDA for DEC plus albendazole could significantly accelerate LF elimination [43]. However, before IDA could be recommended for MDA by WHO, large-scale cohort event monitoring studies were needed to assess tolerability of the treatment in different epidemiological settings. DOLF therefore conducted large community MDA studies in five countries (PNG, Indonesia, Fiji, Haiti, and India) that compared the tolerability of IDA versus DEC plus albendazole in approximately 26,000 participants. No significant differences in adverse event frequency or types were observed after IDA or DEC plus albendazole [44]. The detailed journey of IDA from preliminary clinical trials to a change in WHO MDA guidelines has been described in a series of papers [45,46]. An important component in this journey was a rigorous assessment of IDA acceptability based on a mixed methods protocol followed in each study site [47]. This work culminated in a November 2017 WHO recommendation that endorsed the use (with some qualifications) of IDA for LF MDA for countries that had been using DEC plus albendazole in Africa [23]. That was followed immediately by a commitment by Merck Sharp & Dohme, the manufacturer of ivermectin, to expand its ivermectin donation program by 100 million doses per year for use in LF elimination programs outside of Africa. Some 413 million IDA treatments had been distributed as MDA in 22 countries through the end of 2024 [48].

#### 4.2. IDA implementation research.

While IDA is now widely used for MDA in countries that previously used DEC plus albendazole, a number of important research questions remain. For example, does IDA actually accelerate LF elimination? Implementation research studies also seek to help countries improve MDA strategies, procedures, and practices regarding monitoring and evaluation. Several studies were organized along similar lines, including DOLF studies in Papua New Guinea and Indonesia [49,50]. These studies compared sampling protocols (children versus adults, population-proportional versus risk-adjusted sampling informed by geospatial modeling). Both studies found that adult sampling was much more informative because infections were absent or much less frequent in children than in adults in areas with ongoing transmission. The PNG study (in East New Britain Island province) showed that model-based geospatial sampling was more effective than population proportionate sampling in areas with highly varied LF prevalence [51]. Preliminary results from the East New Britain study suggest that two rounds of IDA with moderate to high compliance are sufficient for LF elimination in most areas, although mop-up activities will be required in areas that had high baseline prevalence.

The Indonesia study (in Belitung district) is being performed in an endemic area for zoophilic *B. malayi* infection. This area was considered to have eliminated LF following 6 rounds of MDA with DEC plus albendazole, because it passed three rounds of transmission assessment surveys. However, post-elimination surveys detected an alarming number of Mf-positive adults [41]. A recent survey in animals showed that macaques and to a lower amount cats and dogs in the area were infected with *B. malayi* [52]. Therefore, an ongoing study is evaluating the impact of two rounds of IDA in this district to test whether animal infections are responsible for resurgence of LF in Belitung. We anxiously await the outcome of these studies. It may be impossible to eliminate LF in areas with zoonotic transmission by MDA alone. As in East New Britain, results from Belitung have clearly shown the importance of sampling adults rather than children for post-MDA surveillance and the superiority of sampling informed by geospatial modeling over population proportionate sampling.

#### 4.3. Moxidectin combination treatments for LF.

Moxidectin is an US FDA-registered drug for treatment of onchocerciasis that leads to a more prolonged clearance of *O. volvulus* microfiladermia compared to ivermectin [53]. Because combined MDA for LF and onchocerciasis is required in co-endemic areas, it was essential to determine whether moxidectin is safe and effective for clearing *W. bancrofti* Mf. This is especially important for the elimination of LF in Africa where onchocerciasis is co-endemic and where IDA triple drug treatment cannot be used. DOLF therefore conducted a clinical trial of moxidectin combination treatments in persons with *W. bancrofti* Mf. This trial showed that moxidectin had no drug–drug interactions with DEC or albendazole in individuals with microfilaremic LF, and that moxidectin combination treatments were as well tolerated as ivermectin combinations [54,55]. In this first trial of moxidectin for LF, moxidectin plus albendazole was dramatically superior to ivermectin plus albendazole for achieving sustained Mf clearance for up to two years and was comparable to triple drug treatments with IDA or moxidectin plus DEC plus albendazole [56]. Ultrasound assessments of motile worms in men showed that moxidectin plus albendazole also had greater macrofilaricidal activity than ivermectin plus albendazole. Given these exciting results, an expert committee recommended collection of large-scale safety data for moxidectin plus albendazole versus ivermectin plus albendazole to support a WHO review of moxidectin for MDA in LF/onchocerciasis coendemic areas. DOLF has partnered with Medicines Development for Global Health (MDGH) and Centre Suisse de Recherches Scientifiques (CSRS) to complete a large-scale safety study in Côte d’Ivoire.

**Significance:** The LF treatment trials were arguably the most impactful studies conducted by DOLF over the past 15 years. Albendazole monotherapy has changed the outlook for LF elimination in central Africa. IDA research led to changes in WHO policy and ivermectin donation that have improved chances for LF elimination outside of Africa. Although more work is needed, moxidectin plus albendazole has the potential to significantly accelerate LF elimination in many endemic countries in Africa that do not have areas with high intensity loiasis. This regimen is highly effective against STH, and it is more effective than ivermectin alone for clearing *O. volvulus* Mf from the skin.

### 5. Clinical development of macrofilaricidal treatments for onchocerciasis

#### 5.1. Assessment of the viability and fertility of adult *O. volvulus* in surgically excised subcutaneous nodules.

Therapeutic trials for onchocerciasis sometimes rely on clearance of skin Mf for efficacy assessments. However, DOLF studies have focused on assessment of macrofilaricidal efficacy (killing or long-term sterilization of adult worms). Although research to identify biomarkers for viable *O. volvulus* worms is currently underway [57], histological assessment of onchocercal nodules that contain adult worms remains the gold-standard approach [58,59]. However, few people have the expertise to analyze nodules, and this is quite labor-intensive. DOLF’s initial trials required shipment of many hundreds of stained microscopy slides for independent, standardized assessments in two locations. Slides were sometimes broken or lost. Therefore, we developed an analysis pipeline whereby stained slides were labeled with barcodes, images were scanned, and high-resolution images were viewed with open-source software [60]. Digital images were shared instead of microscope slides, and readers assessed nodule images without metadata. Digitalization of nodule sections was also a prerequisite for research on using artificial intelligence for nodule assessment outside of DOLF.

#### 5.2. IDA and moxidectin combination treatments for onchocerciasis.

The breakthrough discovery that single-dose IDA treatment of LF results in long-term clearance of microfilaremia raised the question of whether IDA might be safe and effective for other filarial infections, including onchocerciasis. Although a multiple-dose course of DEC was used to treat onchocerciasis prior to the introduction of ivermectin, DEC is no longer used for onchocerciasis because of serious safety concerns. After a careful literature review and formal discussions with experts, DOLF initiated a clinical trial of IDA in persons with onchocerciasis [42]. This study, performed in eastern Ghana, compared safety and macrofilaricidal efficacy of a single dose of IDA, three daily doses of IDA, and ivermectin plus albendazole, the recommended drug regimen for MDA in areas where onchocerciasis is coendemic with LF. Pre-treatment with ivermectin was provided to all study participants before the experimental treatments to minimize the risk of ocular adverse events by reducing intraocular Mf loads [61]. IDA was tolerated as well as ivermectin plus albendazole in this hospital-based clinical trial. Importantly, IDA treatment led to a 40% reduction in the percentage of adult female worms alive and fertile 18 months after treatment relative to the comparator treatment [59].

**Significance:** Onchocerciasis researchers have searched for macrofilaricidal treatments for many decades. A safe and effective macrofilaricide would be as much of a game changer for the global onchocerciasis elimination program as ivermectin was for onchocercal disease control and prevention. DOLF’s IDA study represents a significant advance, because IDA was the first short-course oral treatment shown to have significant (albeit partial) macrofilaricidal efficacy for onchocerciasis [62]. Based on these promising results, we have initiated a new clinical trial in Liberia to compare the macrofilaricidal efficacy of several ivermectin and moxidectin combination treatment regimens (two treatments separated by 6 months).

### 6. Diagnostic and genomic advances

#### 6.1. New antigen and antibody tests for *W. bancrofti.*

The filariasis ICT card test (first marketed by AMRAD ICT in Australia circa 1997) was the first commercial point-of-care test that detected *W. bancrofti* adult worm antigen in human blood [63]. The high sensitivity and convenience of this test made it an important tool for the Global Programme to Eliminate LF.

DOLF researchers used the ICT card test to identify persons with bancroftian filariasis for a planned treatment trial in areas highly endemic for loiasis in the eastern Democratic Republic of the Congo. This study found that persons with positive filarial antigen tests had high *L. loa* Mf counts but no *W. bancrofti* based on PCR analysis. This was the first demonstration that heavy *L. loa* infections sometimes produce false-positive filarial antigen test results [64]. Follow-up studies identified *L. loa* antigens that contain an epitope that is detected by commercial antigen tests for *W. bancrofti* infection [65].

Because of problems with ICT filariasis antigen test after the test production was transferred from Australia to the United States, the Gates Foundation funded production of a second-generation antigen test that is now called the Bioline Filariasis Test Strip (Abbott, Abbott Park, IL) that was simpler to produce, ship, and use and slightly more sensitive than the ICT card test. DOLF (with others) performed initial evaluations of the new test with archived plasma samples and with capillary blood samples in Liberia [66]. DOLF coordinated additional field evaluations of the new test in parallel with the legacy card test in areas with low *W. bancrofti* prevalence following multiple rounds of MDA (in Indonesia and Sri Lanka) and in high prevalence locations in Africa (Liberia, Côte d’Ivoire, Republic of Congo, Democratic Republic of Congo). These studies provided robust data on the performance of the new test [67,68]. WHO subsequently endorsed the switch from the ICT test to the FTS, and GPELF has relied on the FTS for LF mapping and assessing the impact of MDA since that time.

One limitation of filarial antigen testing is that antigenemia often persists for years after Mf have been cleared from the blood following treatment. This can lead to continuation of MDA for years after transmission has been interrupted. DOLF-related studies were performed to address this problem. Our group developed a novel antibody test based on a *W. bancrofti* microfilarial antigen (Wb Bhp1). The antibody test had moderate sensitivity for samples from people with microfilaremia. However, other studies showed that antibody levels to Wb Bhp-1 decrease relatively rapidly after treatments that clear Mf [69,70]. While this test has not yet been commercially developed, characteristics of Wb Bhp-1 suggest that it might have value (perhaps with other antigens) as a serological marker to help guide MDA stopping decisions.

#### 6.2. qPCR for filarial parasites and soil-transmitted helminths.

Quantitative PCR (qPCR) is widely used to amplify and detect parasite DNA in insect vectors and in clinical samples. Prior to DOLF, most projects on *O. volvulus* DNA detection utilized conventional PCR and improved diagnostic tools were needed [71,72]. We used patient samples from DOLF studies to develop and evaluate a new qPCR assay for detection of *O. volvulus* DNA in dried skin samples [73]. We also participated in multicenter studies to develop qPCR-based xenomonitoring of blackflies for *O. volvulus* DNA [74]. Other studies used qPCR-based xenomonitoring of mosquito vectors in Sri Lanka for comprehensive assessment of *W. bancrofti* hotspot areas for programmatic decisions [75,76]. Studies of the effects of MDA for LF on soil-transmitted helminths (STH) in Liberia revealed a disparity between DNA and microscopy results for *Trichuris trichiura* that was due to the similar appearance of *Capillaria* and *Trichuris* ova in Kato-Katz fecal smears [77]. Other studies demonstrated an association between high-level STH infections and alterations in the intestinal microbiome [78].

#### 6.3. Population genomics of *W. bancrofti* and *B. malayi.*

Results from DOLF clinical trials prompted questions regarding whether filarial parasites that were detected after treatment survived treatment or were due to re-infection. To tackle the problem, we developed a protocol for reliable, high-coverage whole-genome sequencing of single Mf for population genomic studies. With samples from our trials in Côte d’Ivoire, we were able to distinguish recrudescence from reinfection with *W. bancrofti* [79]. In related work, we are currently conducting studies to formally demonstrate whether *B. malayi* Mf found in humans and animals in Belitung Indonesia belong to the same parasite population.

**Significance:** Studies reviewed in this section demonstrate the value of vertically integrated parasitology research. Samples from clinical trials are used for basic and translational research that sometimes leads to discoveries that are important for clinical programs. As mentioned in the Introduction, parasitology field work sometimes leads to unexpected or off-target discoveries, and DOLF has certainly had its share of these random but scientifically important events.

### 7. Post-MDA surveillance of LF elimination programs

Washington University researchers worked closely with the Sri Lankan government’s Anti-Filariasis Campaign on post-MDA surveillance of LF elimination. Although the DOLF grant supported much of that work, details regarding those studies are beyond the scope of this review. Briefly, they showed that WHO protocols for verifying LF elimination based on preliminary Mf surveys (so-called “pre-TAS”) and transmission assessment surveys (TAS) [80] were insensitive for detecting areas with ongoing transmission in some post-MDA settings. DOLF studies compared several surveillance approaches (adult TAS, antibody-based TAS in children, and molecular xenomonitoring (MX, detection of filarial DNA in mosquitoes), and found that all of these methods were useful for detecting ongoing transmission in areas that had satisfied WHO criteria for LF elimination according to results of school-based transmission assessment surveys. DOLF studies in Sri Lanka were among the first to use MX at the evaluation unit level, and they showed that MX identified persistent LF hotspots with greater sensitivity and granularity than any of the other approaches. Additional work is needed to address the feasibility and optimal deployment of MX for national LF elimination programs.

Studies performed in other areas reported similar results to those in Sri Lanka. Lessons from these post-MDA studies have been used by experts to develop updated WHO monitoring and evaluation guidelines for the GPELF.

## Concluding remarks

We have summarized several key contributions from the DOLF project in Box 3.

Box 3. DOLF: Key contributions*

1. **Annual versus semiannual MDA for LF and onchocerciasis.** Multiple studies showed little or no benefit of semiannual MDA for LF over annual MDA. Programs should focus resources on providing high-quality annual MDA.
2. **Albendazole MDA is used to eliminate LF in areas with coendemic loiasis**. Repeated rounds of semiannual MDA with albendazole alone can achieve this goal. A more potent purely macrofilaricidal drug combination requiring fewer doses would be of value where drug delivery is logistically challenging.
3. **Effects of community MDA for LF and onchocerciasis on STH prevalence and intensity.** MDA for filarial infections can have dramatic effects on STH, especially hookworm. Additional work is needed regarding strategies to maintain or extend these gains following cessation of MDA.
4. **Clinical development of improved treatments for LF.** IDA and MoxA both represent significant advances that could accelerate LF elimination, especially if the new regimens can be coupled to interventions that increase compliance with MDA.
5. **Clinical development of macrofilaricidal treatments for onchocerciasis.** While triple drug treatment with ivermectin, plus DEC, and albendazole has significantly better macrofilaricidal activity than ivermectin plus albendazole, more work is needed. That is because a well-tolerated and effective macrofilaricide would be a game-changer for onchocerciasis.
6. **The importance of working together.** Great things can happen when researchers and public health program officials join forces. The best advances come from time spent together on the ground, in disease-endemic countries. Data from field studies, especially when unexpected, can be more valuable than results of thought experiments performed by a researcher sitting in a chair in front of a computer.
7. **Vertical integration of research.** This facilitates the bidirectional flow of ideas, samples, and technologies between field sites and research laboratories.

*These conclusions are based on DOLF studies that were performed in particular endemic settings.

Global elimination programs for onchocerciasis and LF are in different stages of completion. While important challenges remain for LF elimination, that program has progressed farther than the parallel program for onchocerciasis [81]. Post-MDA monitoring and evaluation and post-elimination surveillance are more important priorities for LF than a need for better MDA in many countries now that IDA is being used for LF elimination in many countries outside of Africa. MDA with moxidectin plus albendazole could accelerate LF elimination in Africa in areas that are currently using ivermectin plus albendazole.

Elimination of onchocerciasis in Africa with current tools will be a challenge; while local elimination of transmission has occurred in some areas, other foci have significant skin Mf prevalences despite MDA with ivermectin for 20 years or more. New, short-term treatment options with macrofilaricidal or long-term sterilizing effects are needed for onchocerciasis, either for selective treatment or for MDA.

In conclusion, we have summarized how DOLF project research has moved from hypotheses and conjecture to conducting clinical trials that have sometimes led to large-scale field projects and ancillary studies necessary for wide-scale implementation and policy change. It was critically important that DOLF had open lines of communication with WHO (headquarters, regional offices, and country offices), national NTD programs, and other stakeholders to facilitate the bidirectional flow of information. We have also worked closely with social and public health scientists whose insights can shape MDA campaigns to optimize compliance. Successes in highly visible NTD elimination programs may help populations and their political leaders to better appreciate and trust evidence-based public health interventions.

## Supporting information

S1 Table List of the scientific, technical, and management faculty, staff, and advisors of the DOLF consortium (past or present) between November 2009 and May 2025 by affiliation. (DOCX)
