# Supplementary material for: The Death to Onchocerciasis and Lymphatic Filariasis (DOLF) project: Accomplishments and ongoing research
Source: PLoS Negl Trop Dis. 2026 Feb 3;20(2):e0013953. doi: 10.1371/journal.pntd.0013953 (PMC12867263; doi:10.1371/journal.pntd.0013953)
Supplement: S1 Table — (DOCX) [file pntd.0013953.s001.docx]

Supplemental Table 1

List of the scientific, technical and management faculty, staff, and advisors of the DOLF consortium (past or present) between November 2009 and May 2025 by affiliation.

**Washington University School of Medicine, St. Louis Missouri, USA:** Besty Abente, Adebiyi Adeniran, Britt Andersen, Rachel Anderson, Joshua Bogus, Philip J. Budge, Young-Jun Choi, Kurt C. Curtis, Irina Diekmann, Alexandre Dyer, Obiora Eneanya, Joseph Fauver, Nicole Fetcho, Kerstin Fischer, Peter U. Fischer Abakar Gankpala, Charles Goss, Sarah E. Greene, Gina Haida, Augustine Hong, Yuefang Huang, Kassandra Huneycutt, Jennifer Klenke, Pawan Kumar, Jonah Kupritz, Daphne Lew, Melanie M. Lloyd, Andrew Majewski, John Martin, J. Phillip Miller, Makedonka Mitreva, Katiuscia O'Brian, Laura Peer, Ramakrishna Rao, Bruce Rosa, Amy Rush, Lucia Sanchez Di Maggio, Keneth Schechtman, Teresa Tufte, Johnny Vlaminck, Jinli Wangi, Gary J. Weil, Devyn Yates

**Bruyère Research Institute, Ottawa; School of Epidemiology and Public Health, University of Ottawa, Ottawa, Canada:** Alison Krentel, Afzaa Rajabali

**Case Western Reserve University, Cleveland Ohio, USA**: Catherine Bjerum, Krufinta Bun, Dennis Dobbs, Cade Howard, James Kazura, Christopher L. King, Brooke Mancuso, Michael Payne, Jannet Tobon Ramos, Edward Thomsen, Daniel Tisch, Erin Zaletel

**Centers for Disease Control and Prevention, Atlanta, Georgia, USA:** Christine Dubray,

Paul Cantey, Kimberly Won

**Centre de Recherche Clinique de Butembo, Université Catholique du Graben, Site Horizon, Butembo, Democratic Republic of the Congo:** Eric Kanza

**Centre de Recherche en Maladies Tropicales de l’Ituri, Ituri, Congo, Democratic Republic of Congo:** Didier Bakajika

**Centre Suisse de Recherches Scientifiques en Côte d'Ivoire, Abidjan,** **Côte d'Ivoire:** Benjamin Koudou, Olivier Kouadio, Allassane Ouattara, Laurence Yao, Agodio Loukouri

**Department of Infection and Immunity, Papua New Guinea Institute of Medical Research, Goroka and Madang, Papua New Guinea:** Moses Laman, Leanne Robinson

**Department of Parasitology, Faculty of Medicine, Universitas Indonesia, Jakarta, Indonesia:** Taniawati Supali, Iwan Ariwan, Michael Christian, Yenny Djuardi, Yossi Destani, Rahmat Alfian, Elisa Iskander, Noviani Sugianto

**Department of Public Health, Erasmus University Medical Center Rotterdam, The Netherlands:** Wilma Stolk**,** Quirine ten Bosch

**Fred Newton Binka School of Public Health, Univ of Health & Allied Sciences (UHAS), Hohoe/Ho, Ghana:** Nicholas Opoku, Shelter Gordor, Fidelis Anumu, Oscar Debrah, Felix Doe, John Owusu Gyapong

**The George Washington School of Public Health, Washington DC, USA:** Ann Goldman

**GlaxoSmithKline, London, United Kingdom:** Mark Bradley, John Horton

**Fiji Ministry of Health and Medical Services, Suva, Fiji:** Josaia Samuela

**ICMR-Vector Control Research Centre, Puducherry, India:** Purushothaman Jambulingam, Kaliannagounde Krishnamoorthy, Subramanian Swaminathan

**Institute for Medical Microbiology, Immunology and Parasitology, University Hospital Bonn, Bonn, Germany:** Achim Hoerauf, Bettina Dubben, Ute Klarmann-Schulz, Sabine Mand, Arcangelo Ricchiuto, Janina M. Kuehlwein

**Institut de Recherche pour le Développement, INSERM Unité, Université de Montpellier, Montpellier, France:** Michel Boussinesq, Cédric Chesnais, Sébastien Pion, Jeremy Campillo

**Kwame Nkruma University of Science and Technology, Kumasi, Ghana:** Linda Batsa-Debrah, Alexander Y. Debrah

**Liberian Institute for Biomedical Research/National Public Health Institute of Liberia, Charlesville/Monrovia, Liberia:** Fatorma K. Bolay, Patrick Kpanyen, Lincoln Gankpala, Kpehe Bolay, Lawrende Fakoli, Aaron Momolu, Kalilu Donzo, Hayford Howard, Dormu Kollie, Chris Dougbeh Nyan, Cooper Sannah

**Margan Clinical Research Organization (MMARCRO), Accra, Ghana:** Margaret Williams, Michael Nitri

**Medicines Development for Global Health (MDGH)*,* South Bank Victoria, Australia:** Sally Kinrade, Mark Sullivan

**Michigan State University East Lansing Michigan, USA:** Charles MacKenzie

**Ministère de la Santé et de la Population, Brazzaville, Republic of Congo:** Francois Missamou

**Ministère de la Santé et de la Population, Port-au-Prince Haiti:** Jean Frantz Lemoine

**Ministère de la Santé Publique Programme National de Lutte contre les Maladies Tropicales Negligees a Chimiotherapie Preventive (PNLMTN-CTP), Kinshasa, Democratic Republic of Congo:** Naomi Pitchouna Uvon-Awaca

**Ministry of Health, Anti-Filariasis Campaign, Colombo, Sri Lanka:** Sunil Settinayake, Sandhya D. Samarasekera, Devika Mendis, Udaya Ranasinghe, Manjula W. Punchihewa, Indeewarie E. Gunaratna

**National Institutes of Health (NIH), Bethesda Maryland, USA:** Amy Klion

**National Program to Control Schistosomiasis-STH and LF, Abidjan, Côte d'Ivoire:**

Aboulaye Meite

**PanAmerican Clinical Research, Peachtree City, Georgia, USA:** Kathy Lenhard

**Program Studi Ilmu Kesehatan Masyarakat, Program Pascasarjana, Universitas Nusa Cendana, Kupang, Lasiana, Kelapa lima, Kota Kupang, Indonesia:** Adriani Lomiga

**School of Biomedical and Allied Sciences, University of Ghana, Accra, Ghana:** Mahmood A. Seidu, Simon Attah

**St. Thomas Eye Hospital, Accra, Ghana:** Michael Gyasi

**SPRI Clinical Trials Consultants, Brooklyn, New York, USA:** Amy Rigney

**Swiss Tropical & Public Health Institute, Allschwil, Switzerland:** Peter Steinmann, Jürg Ützinger

**Task Force for Global Health, Decatur, Georgia, USA:** Eric Ottesen, David Addiss, Katherine Gass, Adrian Hopkins, Yao Sodahlon, Patrick Lammie, Alex Pavluk

**Tropical Diseases Research Group, Murdoch Children's Research Institute, Melbourne, Victoria, Australia:** Andrew Steer, Myra Hardy

**University of Ruhuna, Galle, Sri Lanka:** Channa Yahathugoda,

Mirani V. Weerasooriya

**World Health Organization, Geneva, Switzerland:** Kazuyo Ichimori, Jonathan King, Antonio Montresor, Maria Rebollo Polo, Tony Ukety
